# Comparison of three-dimensional cell culture techniques of dedifferentiated liposarcoma and their integration with future research

**DOI:** 10.3389/fcell.2024.1362696

**Published:** 2024-03-04

**Authors:** Sayumi Tahara, Soumya Sharma, Fernanda Costas Casal de Faria, Patricia Sarchet, Luisa Tomasello, Sydney Rentsch, Roma Karna, Federica Calore, Raphael E. Pollock

**Affiliations:** ^1^ Department of Surgery, Division of Surgical Oncology, The James Comprehensive Cancer Center, The Ohio State University Wexner Medical Center, Columbus, OH, United States; ^2^ Department of Cancer Biology and Genetics, The James Comprehensive Cancer Center, The Ohio State University Wexner Medical Center, Columbus, OH, United States

**Keywords:** three-dimensional (3D) model, dedifferentiated liposarcoma, collagen, hanging drop, Matrigel, ULA plate, microfluidic device, spheroid

## Abstract

**Background:** Dedifferentiated liposarcoma is a formidable sarcoma subtype due to its high local recurrence rate and resistance to medical treatment. While 2D cell cultures are still commonly used, 3D cell culture systems have emerged as a promising alternative, particularly scaffold-based techniques that enable the creation of 3D models with more accurate cell-stroma interactions.

**Objective:** To investigate how 3D structures with or without the scaffold existence would affect liposarcoma cell lines growth morphologically and biologically.

**Methods:** Lipo246 and Lipo863 cell lines were cultured in 3D using four different methods; Matrigel^®^ ECM scaffold method, Collagen ECM scaffold method, ULA plate method and Hanging drop method, in addition to conventional 2D cell culture methods. All samples were processed for histopathological analysis (HE, IHC and DNAscope™), Western blot, and qPCR; moreover, 3D collagen-based models were treated with different doses of SAR405838, a well-known inhibitor of MDM2, and cell viability was assessed in comparison to 2D model drug response.

**Results:** Regarding morphology, cell lines behaved differently comparing the scaffold-based and scaffold-free methods. Lipo863 formed spheroids in Matrigel^®^ but not in collagen, while Lipo246 did not form spheroids in either collagen or Matrigel^®^. On the other hand, both cell lines formed spheroids using scaffold-free methods. All samples retained liposarcoma characteristic, such as high level of MDM2 protein expression and *MDM2* DNA amplification after being cultivated in 3D. 3D collagen samples showed higher cell viability after SAR40538 treatment than 2D models, while cells sensitive to the drug died by apoptosis or necrosis.

**Conclusion:** Our results prompt us to extend our investigation by applying our 3D models to further oncological relevant applications, which may help address unresolved questions about dedifferentiated liposarcoma biology.

## 1 Introduction

Liposarcoma (LPS) is the most common histological type of soft tissue sarcoma, and dedifferentiated liposarcoma (DDLPS) is a subtype that exhibits aggressive clinical behavior, with a high propensity for local recurrence and metastatic potential (Thway, 2019). Although traditional two-dimensional (2D) cell cultures still play a pivotal role in several research fields (Duval et al., 2017), these fail to accurately mimic the *in vivo* microenvironment (Zanoni et al., 2020), and recent focus has therefore shifted towards three-dimensional (3D) culture systems.

3D cell culture techniques are broadly classified as scaffold-based or scaffold-free. The scaffold-based technique provides an environment for cell cultures that is similar to *in vivo* biological conditions (Duval et al., 2017). Matrigel is one of the most common materials for organoid cultivation, which is prepared from the secretion of Engelbreth-Holm-Swarm mouse sarcoma cells. Similar techniques have been employed to create simulated models of the intestine (Sato et al., 2009), heart (Nugraha et al., 2019; Miyamoto et al., 2021), brain (Wang, 2018; Sidhaye and Knoblich, 2021), liver (Ogoke et al., 2021), kidney (Yousef Yengej et al., 2020; Shimizu et al., 2021) and pancreas (Balak et al., 2019). However, due to the extreme complexity of Matrigel which consists of more than 1,800 unique proteins (Hughes et al., 2010), the undefined components make it difficult to identify the signals required for organoid structure and function. Collagen, in contrast, is commonly used as a scaffold since it is a primary component of the extracellular matrix (ECM) (Perez-Puyana et al., 2020). Moreover, collagen scaffold impact on cell migration, cellular adhesion, proliferation, and differentiation has been reported in numerous cell types; also, collagen scaffold contains a porous surface whose size is adjustable through the manipulation of ionic force, pH, temperature, and collagen concentration, that enables to generate the desirable condition for the specific functions and properties of tissues (Habanjar et al., 2021). Therefore, this unique characteristic of collagen allows to facilitate 3D cell culture optimization and many collagen-based 3D models of *in vitro* cancer culture have been developed as a result.

The scaffold-free techniques can also facilitate cell-cell communication and possibly provide results more resembling to *in vivo* condition than conventional cell culture. Scaffold-free techniques minimize the time to form 3D structures compared to scaffold-based techniques, thanks to simple surrounding environment as there are no other biomaterials used to support cell growth, hence requiring less time to adjust (Alblawi et al., 2020). Moreover, Sun et al., 2022 suggested that scaffold-free tissue constructs could be superior to scaffold-based constructs because the produced matrix was relying only on cells. Therefore, no interference between cell-cell interaction or cell migration as scaffold-free techniques do not require cell adherence or exogenous materials (Leight et al., 2012). However, Alghuwainem A et al. advocated some concerns about scaffold-free structures and these definitions, an attempt to develop a replica mimicking living tissue using only cells remains questionable (Alghuwainem et al., 2019).

Our previous study showed that scaffold existence could affect morphological structures, and 3D models demonstrated different drug tolerance compared to 2D models using our Lipo246 and Lipo863 cell lines (Tahara et al., 2023). In this study, we established two new techniques for creating 3D models of liposarcoma: collagen embedded method and hanging drop method in addition to the Matrigel^®^ method and Ultra-low attachment (ULA) plate method. Our goal was to assess whether the presence of a scaffold could affect liposarcoma cell lines morphologically and biologically. All methods were subjected to histopathological assessment, Western blot (WB), and real-time PCR (qPCR) analysis. The collagen 3D models were also utilized for cell viability and apoptotic assessment after drug treatment in order to compare the differences in cell survivability between conventional 2D models, 3D structures and the presence of a scaffold. The presence of a scaffold and the selection of a specific 3D cell culture method may affect protein and gene expression. For this reason, research projects should be designed accordingly depending on the final purpose (Mooney et al., 1997; Carletti et al., 2011). We believe our results will positively contribute to future investigations, involving the use of microfluidic devices, and provide further knowledge for a better understanding of liposarcoma pathobiology.

## 2 Materials and methods

### 2.1 Cell lines and two-dimensional (2D) cell culture

Human liposarcoma cell lines Lipo141, 224, 246, 815, and 863 were established in our laboratory as previously described (Peng et al., 2011). Lipo246 were grown in Dulbecco’s Modified Eagle’s Medium (DMEM)/F12 (Gibco) supplemented with 10% FBS. The other cell lines, Lipo141, 224, 815, and 863 were grown in DMEM (Gibco) supplemented with 10% (vol/vol) fetal bovine serum (FBS) from Gibco. All cells were maintained in 5% CO_2_ at 37°C and tested for *mycoplasma*.

### 2.2 Three-dimensional (3D) cell culture

#### 2.2.1 Matrigel^®^ ECM scaffold method

**FIGURE 1:**
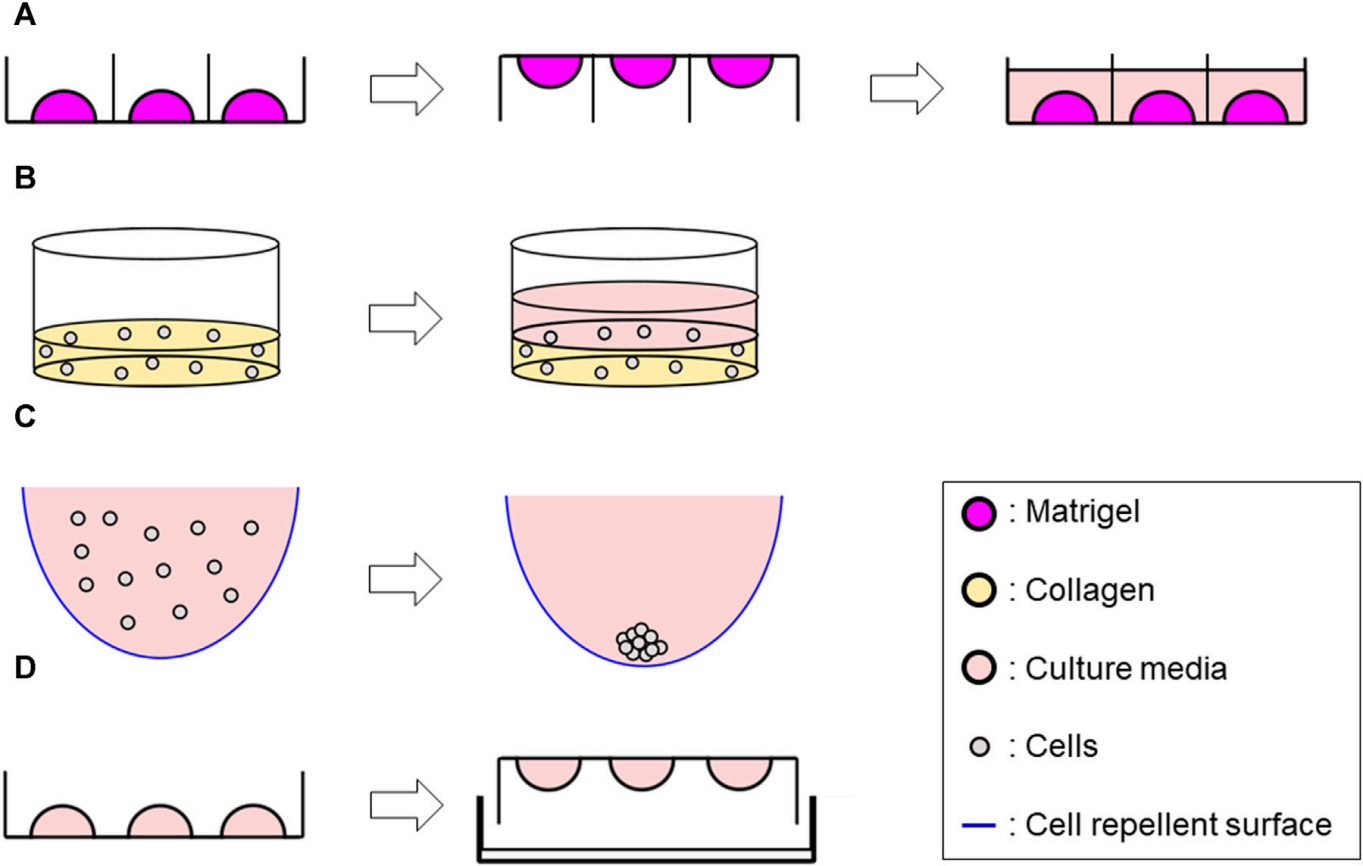
Schematic representation of 3D methods used in this study. **(A)** Matrigel^®^ ECM scaffold method Generate 50 μL of Matrigel^®^/cell suspension mix dome → flip the plate after 3 min incubation → reverse the plate after 15–20 min incubation, then add 500 μL of culture media along the wall. **(B)** Collagen ECM scaffold method (layer) Seed 1 mL/well of the mix of single-cell suspension and collagen solution into a 12-well plate and incubate at 37°C for 30 min to solidify the mixture–> add 1 mL of culture media. **(C)** ULA plate method Add 200 μL of cell suspension per well in a ULA plate (96 well-plate) whose wall was previously covered with a cell repellent solution. **(D)** Hanging drop method Develop 10 µL drops of cell suspension on an inverted 60 mm tissue culture dish lid–> add DPBS on the dish bottom, then integrate with the lid in the right-side-up orientation.

Cells were seeded into Matrigel^®^ (Corning, Cat # CLS354234) as previously reported (Tahara et al., 2023). Briefly, a mixture of 50 μL Matrigel^®^/cell mixture containing 4 × 10^3^ single cells was formed into a dome shape in a 24-well plate. Once the dome was incubated for 3 min at 37°C, the plate was flipped upside down and incubated for additional 15–20 min. The plate was finally returned to a right-side-up orientation and 500 μL of culture media per well were added. Plates were incubated at 37°C, and the growth medium was changed every 2–3 days. Cultures were maintained up to 14 days (Figure 1A).

#### 2.2.2 Collagen ECM scaffold method

A Type I collagen-based hydrogel solution was prepared by mixing Rat tail collagen type I (CORNING, Cat #354236) with ×10 Dulbecco’s phosphate-buffered saline (DPBS) (Gibco, Cat #14080055), 1N NaOH (AMRESCO, Cat #1310-73-2) and double-distilled sterile water to yield an ECM solution with final concentrations of 3 mg/mL collagen with a pH of 7.4, and ×1 DPBS on ice. Cell suspension (1 × 10^5^ cells/mL) and the collagen solution were then mixed with a 1:1 ratio on ice. For Collagen layer method, 1 mL/well of the mixture was seeded into a 12-well plate, while 50 µL of the mixture was seeded into a 24-well plate for Collagen droplet method. The plate was incubated at 37°C for 30 min to solidify, then 1 mL of culture media for Collagen layer method or 500 µL of culture media for Collagen droplet method were added to each well. Plates were incubated at 37°C and the growth medium was changed every 2–3 days up to 14 days (Figure 1B; Supplementary Figure S1).

#### 2.2.3 Ultra-low attachment (ULA) plate method

200 µL of cell suspension (8 × 10^4^ cells/mL) were seeded in 96-well round-bottom ultra-low attachment plates (CORNING, Cat #7007) and incubated up to 72 h, then processed for further analyses (Figure 1C).

#### 2.2.4 Hanging drop method

10 µL drops of cell suspension (2.5 × 10^6^ cells/mL) were developed on an inverted 60 mm tissue culture dish lid, with approximately 20–30 drops per dish. DPBS was added to the dish bottom to prevent dehydration. The lid was returned to the right-side-up orientation and integrated to the bottom part. The entire plate was incubated for 72 h at 37°C, then further analyses were performed (Figure 1D).

All images were taken using an EVOS XL Core Imaging system (Life Technologies).

### 2.3 Histopathological analysis (HE staining, IHC and DNAscope™)

For scaffold-based models (Matrigel^®^ and collagen embedded), supernatant media was aspirated from the plate, Matrigel^®^ domes and collagen layers were stained with a 1:1 solution of Hematoxylin: PBS for 10 s, then the stained samples were embedded into a liquified 2% agarose gel solution. For scaffold-free models (ULA plate and Hanging drop), samples were collected into microtubes, stained with Hematoxylin: PBS solution, washed with PBS, then embedded in liquified 2% agarose gel solution. Samples were placed at 4°C for 15 min to solidify agarose gel completely and following to be fixed with 4% paraformaldehyde for 48–72 h.

After fixation, samples were paraffin-embedded and sliced into 10 μm (for scaffold-based samples) or 4 µm (for scaffold-free samples) sections for Hematoxylin and eosin (HE) staining, immunohistochemistry (IHC) and DNAscope™ analyses. For IHC, antibodies for Murine Double Minute 2 (MDM2) (Thermo Fisher Scientific, Cat# 182403, RRID:AB 2920937) and Ki-67 (Dako, Cat# M7240) were used. Positive staining was visualized with diaminobenzidine (DAB), and counterstained with hematoxylin. Ki-67 positive index was calculated using microscope. DNAscope™ is a method designed for the detection of DNA molecules specifically in paraffin-embedded tissue slides. For this study, we used the probe for *MDM2* named DS-Hs-MDM2-C1 (Advanced Cell Diagnostics) which was designed to target the region chr12:68808149-68850687. DNAscope™ was performed following manufacturer’s instructions and described previously (Casadei et al., 2022). All images were taken using a Axioskop 40 FL (Zeiss).

### 2.4 Cell recovery from scaffold-based method

#### 2.4.1 Matrigel^®^

Corning^®^ Cell Recovery Solution enabled us to retrieve cells from Matrigel^®^. Matrigel^®^-embedded samples were placed at 4°C for 30 min allowing Matrigel^®^ to be liquified. All samples were transferred into new tubes with existent growth media and centrifuged at 1,200 rpm for 3 min at 4°C, then the supernatant was removed leaving only the Matrigel^®^ layer including cells. An appropriate amount of Cell Recovery Solution (CORNING, Cat #354253) was next added to the Matrigel^®^/cell mix (the amount of the solution was recommended to be ≧2x that of Matrigel volume), which was then incubated at 4°C overnight. Cells were centrifuged at 1,200 rpm for 3 min at 4°C, and supernatant was aspirated. 1 mL of cold (4°C) PBS was added to the remaining cell pellet, mixed gently, then transferred into a microtube and centrifuged at 5,000 rpm for 3 min. After discarding the supernatant, cells were washed with cold PBS. Finally, cells were centrifuged at 15,000 rpm for 15 min, the supernatant discarded, and purified cell pellet was collected.

#### 2.4.2 Collagen

Samples were collected in a tube containing DMEM supplemented with 5% FBS and 100 µL of 3% collagenase and incubated at 37°C until collagen was completely degraded. Samples were then centrifuged at 1,200 rpm for 3 min at room temperature and washed with PBS twice, then retrieved cell pellets were collected.

### 2.5 Western blot analysis

For Western blot analysis, cells were lysed with ice-cold Cell Lysis Buffer (Cell Signaling, Cat #9803S) supplemented with Halt™ Protease and Phosphatase inhibitor Cocktail (Thermo Scientific, Cat #1861281) and incubated at 4°C for 30 min to extract proteins. Equivalent amounts of proteins were first mixed with sample buffer (Bio-Rad, Cat #1610747), then loaded on a Tris-HCl 4%–20% precast polyacrylamide gel (Bio-Rad, Cat #4561094) and transferred onto nitrocellulose membranes (Bio-Rad, Cat #1704159). Membranes were probed overnight at 4°C with anti-MDM2 (Cell Signaling Technology Cat# 86934, RRID:AB_2784534) and anti-GAPDH antibodies (Santa Cruz Biotechnology Cat# sc-48167, RRID:AB_1563046), followed by incubation with fluorescence-conjugated secondary antibodies IRDye^®^ 800CW (Li-Cor, Cat #926-32213, RRID: AB_621848) and IRDye^®^ 680RD (Li-Cor, Cat #926-68074, RRID: AB_10956736). The proteins of interest were finally detected using Odyssey CLx Imaging System (Li-Cor). The band density of proteins was quantified using densitometric Image Studio Software Ver. 5.2 (Li-Cor Biosciences).

### 2.6 *MDM2* DNA quantification and qPCR analysis

Total DNA was isolated from cell lines or 3D models by using QIAamp^®^ DNA Mini Kit (QIAGEN, Cat #51304) following the manufacturer’s instructions. Purified genomic DNA sample concentration and quality were assessed using NANODROP ONE^C^ (Thermo scientific). For the determination of *MDM2* DNA expression levels (for both cell lines and 3D models) via real-time PCR, 20 ng of DNA were used per reaction and DNA sequence-specific probes for MDM2 (Thermo Fisher, Cat #Hs0054450_s1) and GAPDH (Thermo Fisher, Cat # Hs03929097_g1) were used.

### 2.7 Drug response assay

Drug response was assessed using a cell viability/cytotoxicity assay as well as an Annexin V/PI assay using Lipo246 and Lipo863 collagen 3D models compared to conventional 2D cell culture methods. Collagen 3D models were prepared following the process described above. For 2D cell cultures, 3 × 10^5^ Lipo246 cells or 1 × 10^5^ Lipo863 cells were plated to each well of a 12-well plate. The following day, cells were treated with DMSO (control) or increasing doses of MDM2 inhibitor SAR405838 (Sigma, SML2772) (0.1, 0.5, 1, 2, 5 µM) up to 72 h. The efficacy of SAR405838 on Lipo246 and Lipo863 cell lines was previously evaluated by our group (Bill et al., 2016).

#### 2.7.1 Cell viability assay

The effects of SAR40538 on the viability of Lipo246 and Lipo863 cells was evaluated using Invitrogen™ LIVE/DEAD™ Viability/Cytotoxicity Kit for mammalian cells (Invitrogen, Cat #L3224) following the manufacture’s protocol. After 72 h of incubation with the drug, cells were washed, resuspended in 1.0 mL of PBS, and then 2 µL of 50 µM calceinAM working solution and 4 µL of ethidium homodimer-I were added. Cells were incubated for 20 min prior to viability assessment by flow cytometry (LSRFortessa, BD Biosciences) analysis, using 488 nm excitation. Results were analyzed using FlowJo_v10.6.1 software. Cells were gated excluding auto fluorescent cells according to negative control stains.

#### 2.7.2 Apoptosis analysis by annexin V-FITC/PI staining

The number of apoptotic and necrotic cells was assessed using TACS^®^ Annexin V-FITC Apoptosis Detection kit (R&B System, Cat #4830-250-K) according to the manufacturer’s protocol. Cells were washed with DPBS, and each sample was resuspended in 100 µL of Annexin V Incubation Reagent obtained by combing 10 µL of ×10 Binding Buffer, 10 µL of Propidium Iodide (PI), 1 µL of TACS Annexin V-FITC and 79 µL of distilled water. After 15 min of incubation at room temperature, protected from light, the apoptotic and necrotic cell percentages were then immediately assessed via cytometry (LSRFortessa, BD Biosciences) analysis, using 488 nm excitation, and data were analyzed using FlowJo_v10.6.1 software.

### 2.8 Statistics

All experiments were carried out as three independent experiments and results were represented as mean ± SD. Student’s *t*-tests or one-way ANOVA was conducted to conclude statistical significance: differences were considered being significant at *p* < 0.05, using GraphPad Prism version 9.2.0 software.

### 2.9 Ethics approval

This study did not involve participation of human subjects or use of animals.

## 3 Results

### 3.1 DDLPS cells were successfully cultivated in 3D using all methods

**FIGURE 2:**
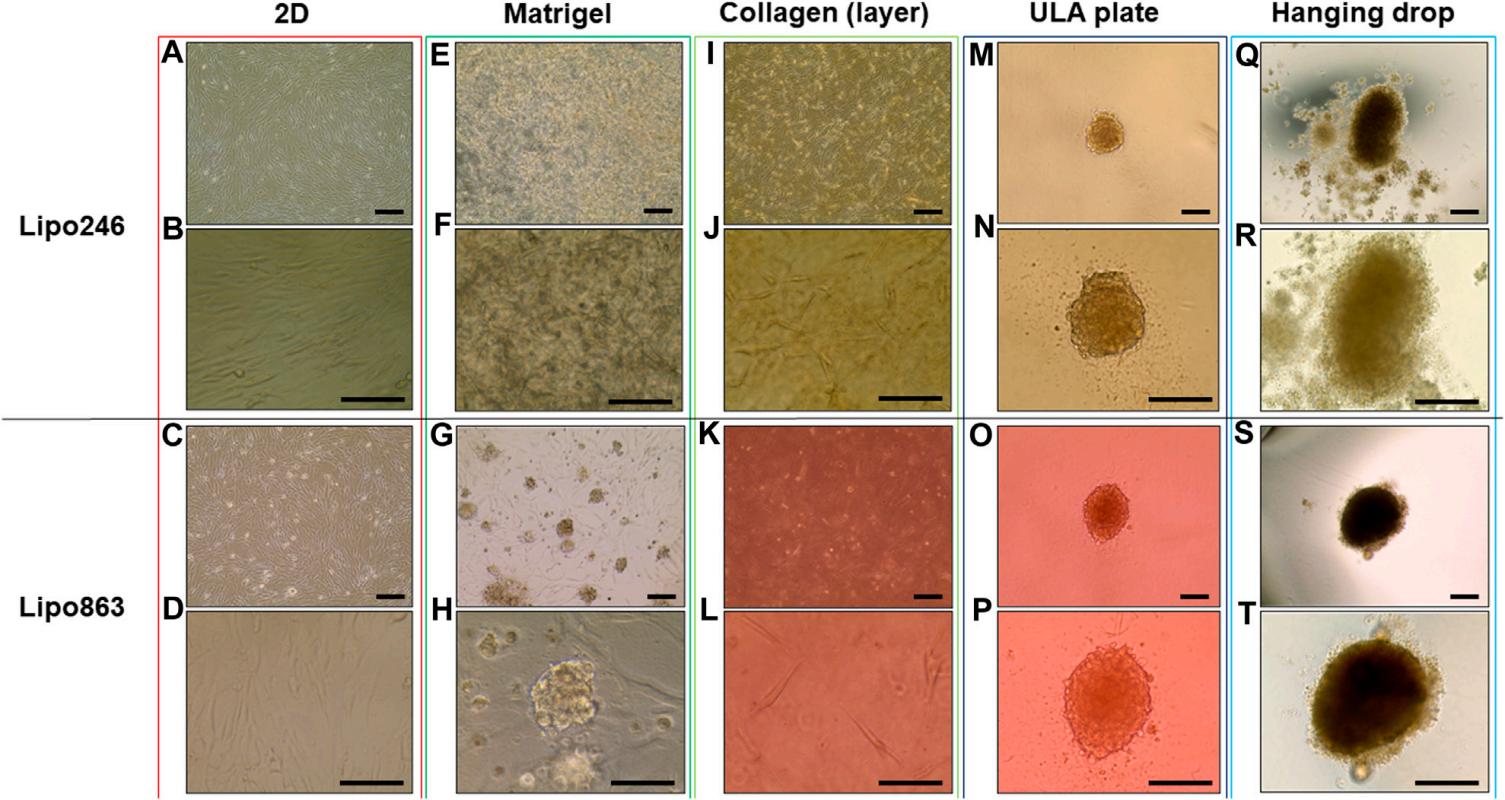
Representative images of Lipo246 and Lipo863. Lipo246 and Lipo863 grown in 2D cell culture, and four different 3D cell culture methods were imaged. **(A–D)** 2D cell culture, **(E–H)** Matrigel^®^ ECM scaffold method 3D cell culture, **(I–L)** Collagen ECM scaffold method 3D cell culture, **(M–P)** ULA plate method 3D cell culture and **(Q–T)** Hanging drop method 3D cell culture of Lipo246 and Lipo863, respectively. Images were taken with ×10 objectives **(A,C,E,G,I,K,M,O,Q,S)**, ×20 objectives **(N,P,R,T)** or ×40 objectives **(B,D,F,H,J,L)**. Scale bar is indicating 250 μm in **(A,C,E,G,I,K,M–T)**, and 125 µm in **(B,D,F,H,J,L)**.

All samples successfully generated 3D structures from liposarcoma cell lines. Both Lipo246 and Lipo863 showed spindle-shaped cells distributed in loose whorls in conventional cell culture; no significant morphological differences were detected among them (Figures 2A–D). However, some differences were revealed when cells were cultivated using scaffold-based techniques. Lipo863 formed spheroids in Matrigel^®^ ECM scaffold, but not in collagen layer (Compare Figures 2G, H with Figures 2K, L). Lipo246, on the other hand, did not generate spheroids either in Matrigel^®^ or collagen ECM scaffold (Figures 2E, F, I, J), and cells spread out radially within scaffold where every single cell was bridging each other. Next, to investigate whether cell’s ability to form spheroids was affected by the type of scaffold, not by the volume or shape of scaffold itself, we generated Collagen ECM droplet model using the same two cell lines. Results showed that neither Lipo246 nor Lipo863 formed spheroid even though the collagen droplets shape was similar to the Matrigel^®^ ECM model domes (Supplementary Figure S2). Moreover, to verify the reproducibility of this result, other three liposarcoma cell lines, Lipo141, Lipo224 and Lipo815, were added and cultivated using Matrigel ECM, Collagen ECM scaffold layer and Collagen ECM scaffold droplet methods. All of the cell lines formed spheroids in Matrigel^®^, while none of them formed spheroid in either Collagen layer or droplet (Supplementary Figure S3). The whole spheroid formation ability comparison was summarized in Table 1. In conclusion, the type of scaffold affected the cell’s spheroid formation ability. In contrast, both Lipo246 and Lipo863 cell lines formed spheroids using scaffold-free techniques, such as ULA plate and hanging drop (Figures 2M–T). Even though the results appeared similar between these two methods, differences were observed. First, ULA plates required a shorter incubation time to generate spheroids (around for 24–48 h) compared to the hanging drop method (around up to 72 h). Second, single spheroids were made in each well of ULA plates, and all spheroids looked unvarying. In contrast, spheroids obtained using hanging drop technique were not as consistent as those seen in ULA plate, and sometimes several spheroids were generated within one droplet. Third, ULA plate spheroids were the same size and quite uniformed through the entire plate, whereas spheroids obtained by hanging drop method had more variety in size. Moreover, the surface of spheroids in ULA plate was smoother and cells were more compactly aggregated than those obtained with the hanging drop protocol. Even though spheroid generation was successfully accomplished, a better understanding about the characteristics of spheroids from each method will help in designing future investigations. Considering these morphological features, Lipo246 and 863 were selected as representative cell lines from now. Furthermore, since the Collagen layer method and the Collagen droplet method showed the same results among 5 cell lines, Collagen layer models were chosen for further analyses.

**TABLE 1:** Summary of spheroid formation ability for five DDLSP cell lines across five different 3D cell culture methods.

	Lipo141	Lipo224	Lipo246	Lipo815	Lipo863
Matrigel (droplet)	+	+	−	+	+
Collagen layer	−	−	−	−	−
Collagen droplet	−	−	−	−	−
ULA plate	N/A	N/A	+	N/A	+
Hanging drop	N/A	N/A	+	N/A	+

### 3.2 Histopathological analysis

**FIGURE 3:**
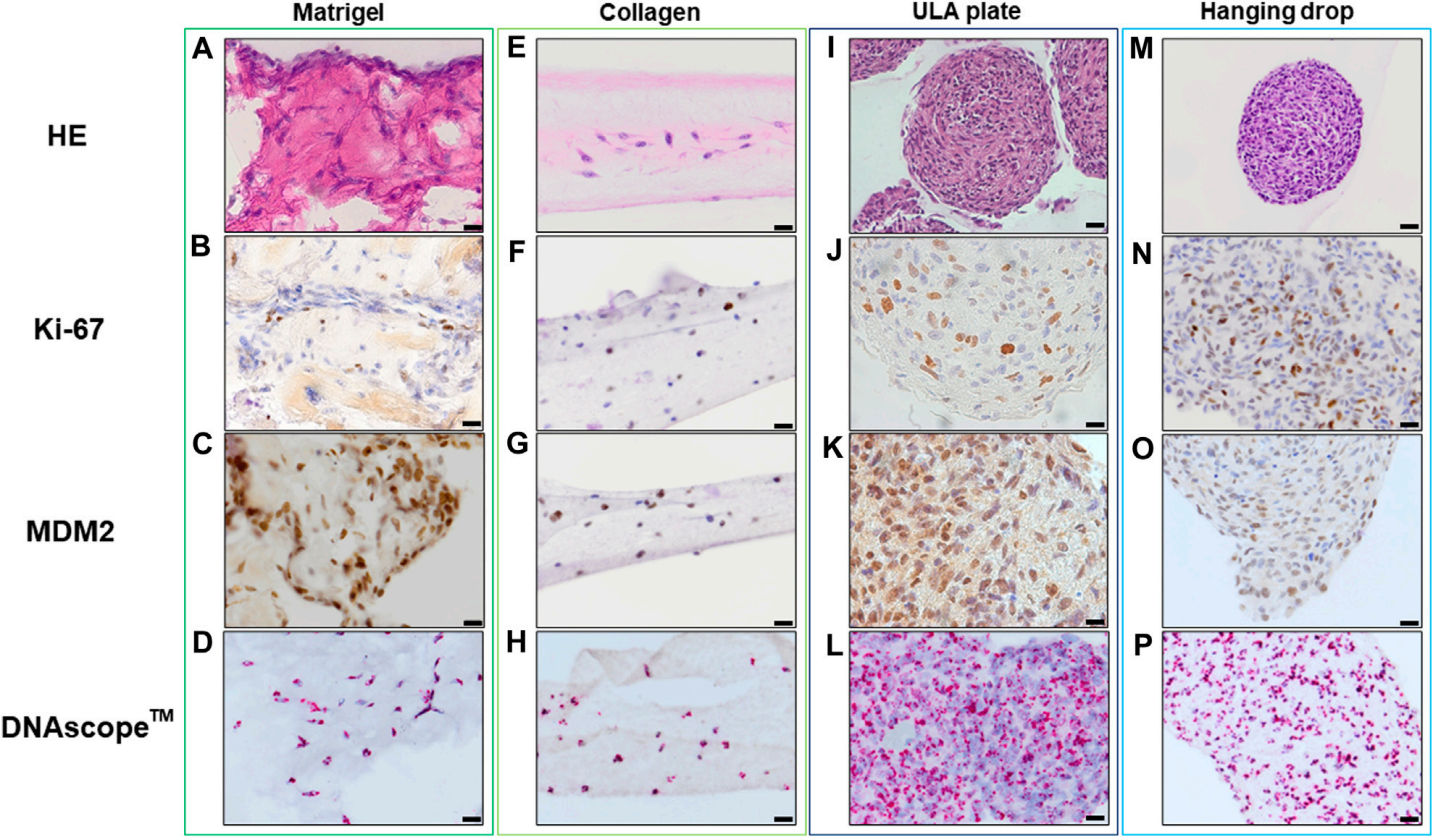
HE, IHC and DNAscope™ images of 3D cell culture constructs (Lipo246). Lipo246 3D models obtained using Matrigel^®^ ECM scaffold. **(A–D)**, Collagen ECM scaffold **(E–H)**, ULA plate **(I–L)** and Hanging drop **(M–P)** methods were stained with HE or with antibodies for Ki-67 and MDM2 for IHC analysis. DNAscope™ was used for *MDM2* DNA detection. The brown precipitation in IHC is indicating the presence of the target antigen. In DNAScope analysis, amplified *MDM2* DNA is visualized as red dots. Scale bars indicate 40 µm **(I,M)** or 20 μm **(A–H,J–L,N–P)**.

**FIGURE 4:**
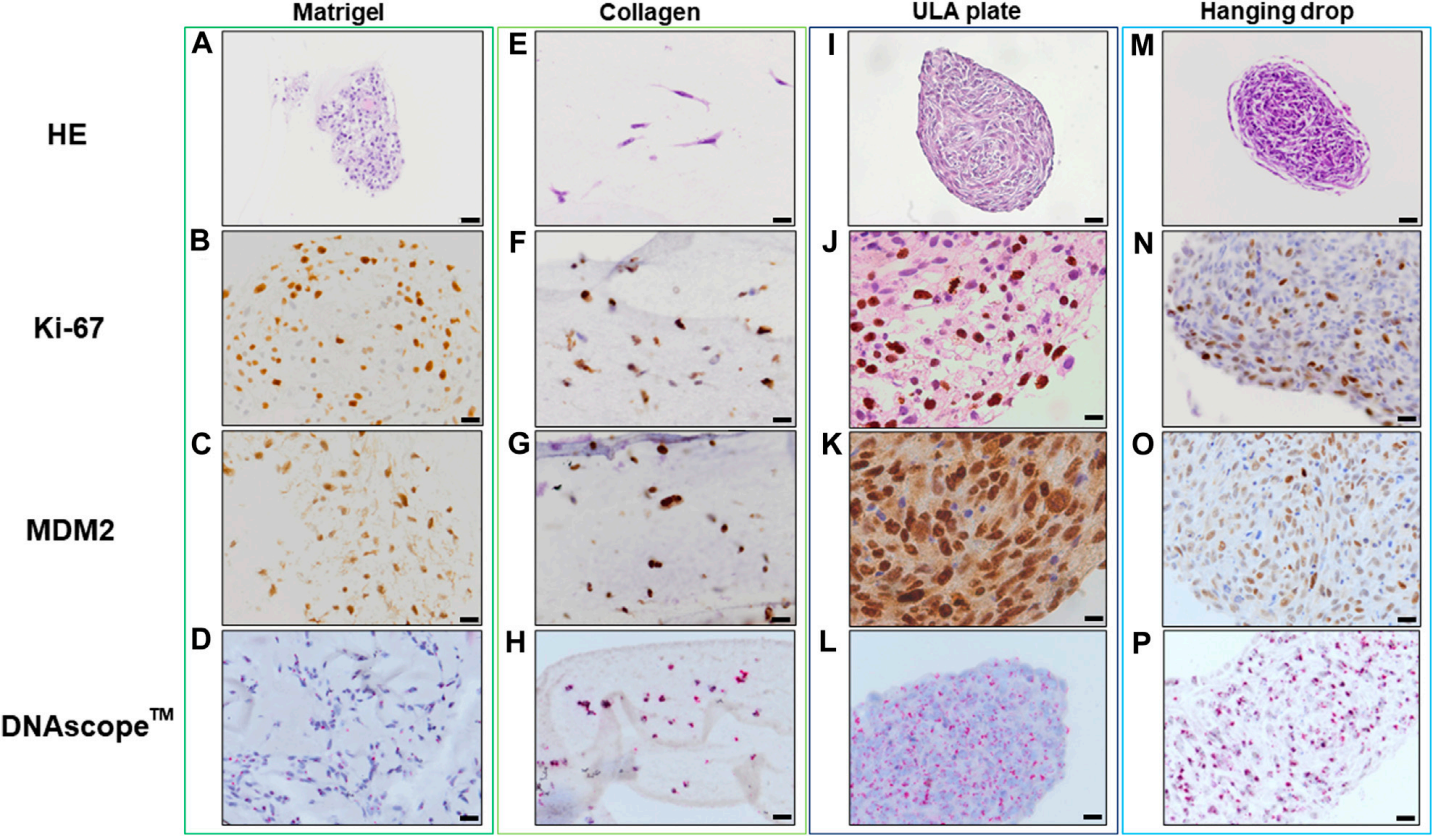
HE, IHC and DNAscope^TM^ images of 3D cell culture constructs (Lipo863) Lipo863 3D models obtained using Matrigel^®^ ECM scaffold. **(A–D)**, Collagen ECM scaffold **(E–H)**, ULA plate **(I–L)** and Hanging drop **(M–P)** methods were stained with HE or with anti-Ki-67 and anti-MDM2 antibodies for IHC analysis. DNAscope^™^ was used for *MDM2* DNA detection. The brown precipitation in IHC is indicating the presence of the target antigen. Amplified *MDM2* DNA is visualized as red dots. Scale bars indicate 40 µm **(I,M)** or 20 μm **(A–H,J–L,N–P)**.

Histopathological analyses were compatible with the result displayed in Figure 2, spheroid formation was confirmed in ULA plate and hanging drop method for Lipo246 (Figures 3I, M), and Matrigel^®^, ULA plate and hanging drop methods for Lipo863 (Figures 4A, I, M). Lipo863 spheroids from scaffold-free techniques showed higher cellularity than spheroids derived from Matrigel^®^. Single cell distribution within ECM scaffold material was confirmed in collagen method for Lipo246 and Lipo863 (Figure 3E, Figure 4E), and Matrigel^®^ method for Lipo246 (Figure 3A). All cells were spindle-shaped and sometimes bridging to each other. Nucleoli and mitotic figure were also sparsely observed. Ki-67 cell proliferation index for Lipo246 cells was 20% (Figure 3B), 26% (Figure 3F), 37% (Figure 3J), 44% (Figure 3N), whereas for Lipo863 the proliferation index was 67% (Figure 4B), 66% (Figure 4F), 37% (Figure 4J), 31% (Figure 4N), respectively. MDM2 protein expression and *MDM2* DNA amplification were confirmed by IHC for both Lipo246 cells (Figures 3C, G, K, O) and Lipo863 cells (Figures 4C, G, K, O) as well as by DNAscope™ (for Lipo246, Figures 3D, H, L, P, and for Lipo863 Figures 4D, H, L, P). In DNAscope™ analysis, every single red dot indicates the presence of target gene *MDM2* amplification. Regardless of the culture method, all liposarcoma models were diffusely or mostly positive for MDM2 staining in IHC and *MDM2* DNA detection in DNAscope™.

### 3.3 MDM2 protein could be successfully detected in 3D constructs established with all the tested methods

**FIGURE 5:**
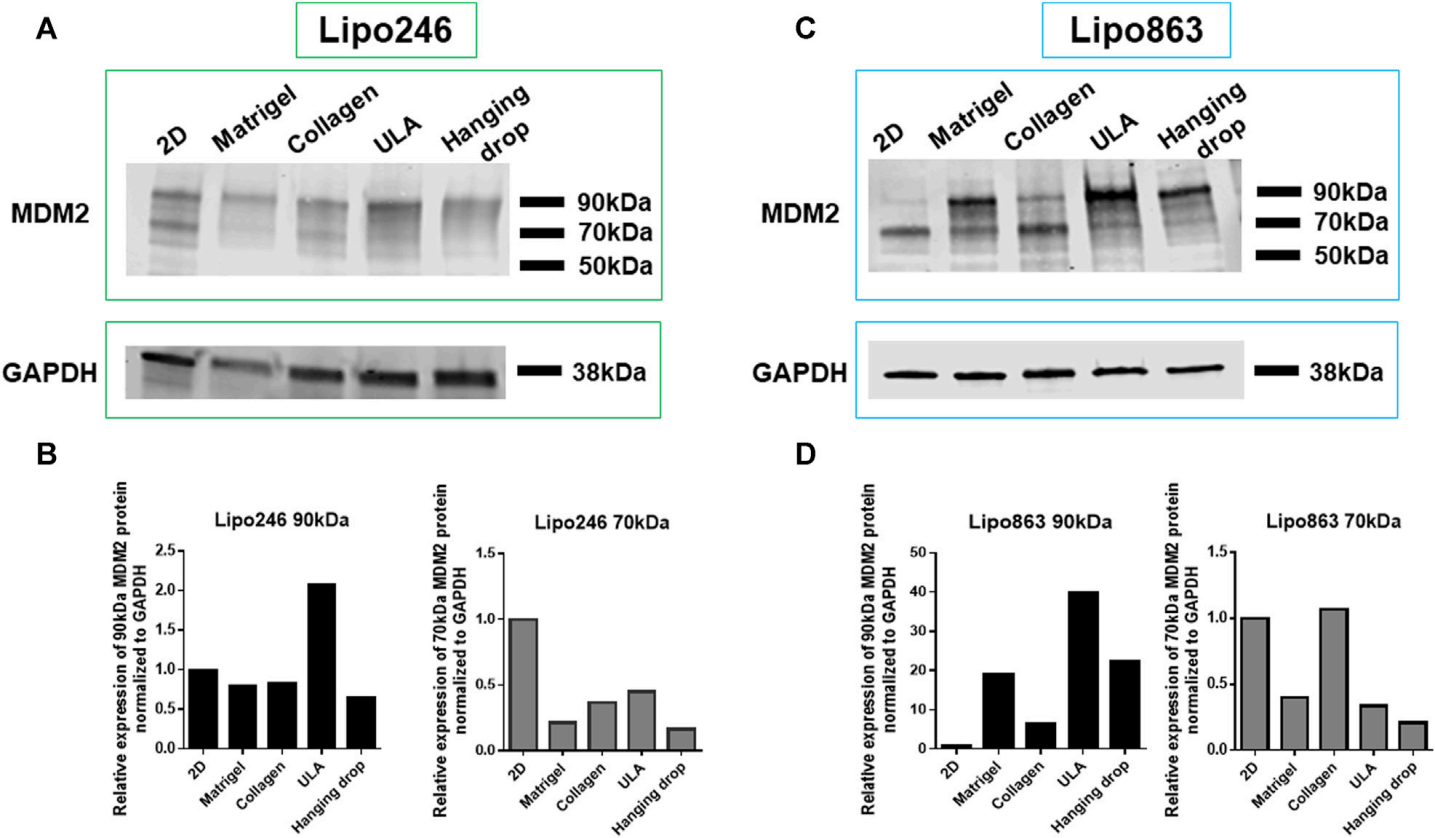
MDM2 protein expression assessment by Western blot. WB analysis showing MDM2 protein expression for conventionally cultured Lipo246 and 863 and for all 3D methods. Results for Lipo246 are shown in **(A)**, while densitometry analysis was performed for MDM2 full-length [**(B)**, 90 kDa, black bars] and for MDM2 isoform [**(B)**, 70 kDa, gray bars] normalized to GAPDH. Results for Lipo863 are indicated in **(C)** and densitometry analysis is shown in **(D)** for MDM2 full length (black bars) and for the 70 kDa isoform (gray bars) normalized to GAPDH.

Lipo246 and Lipo863 cells cultivated both in 2D and 3D techniques were processed for Western blot analysis to assess the expression levels of MDM2 protein as shown in Figure 5. MDM2 (whose full-length corresponds to 90 kDa) was detected in all samples for both cell lines, supporting the idea that MDM2 amplification, the main dedifferentiated liposarcoma feature, was still preserved in 3D cultures (Figure 5). At the same time, an additional band of 70 kDa, corresponding to an MDM2 isoform, was detected within most samples. In both Lipo246 and Lipo863, the stronger expression of the 90 kDa protein was detected when ULA plates were used. In Lipo246, the 70 kDa isoform was highly expressed when cells were cultivated using the conventional method compared to all 3D models; whereas in Lipo863 the protein was well-detected both in 2D and collagen cultivated cells. We are currently performing further investigations to elucidate the role of different MDM2 isoforms.

### 3.4 *MDM2* DNA expression was consistent in all 3D models

**FIGURE 6:**
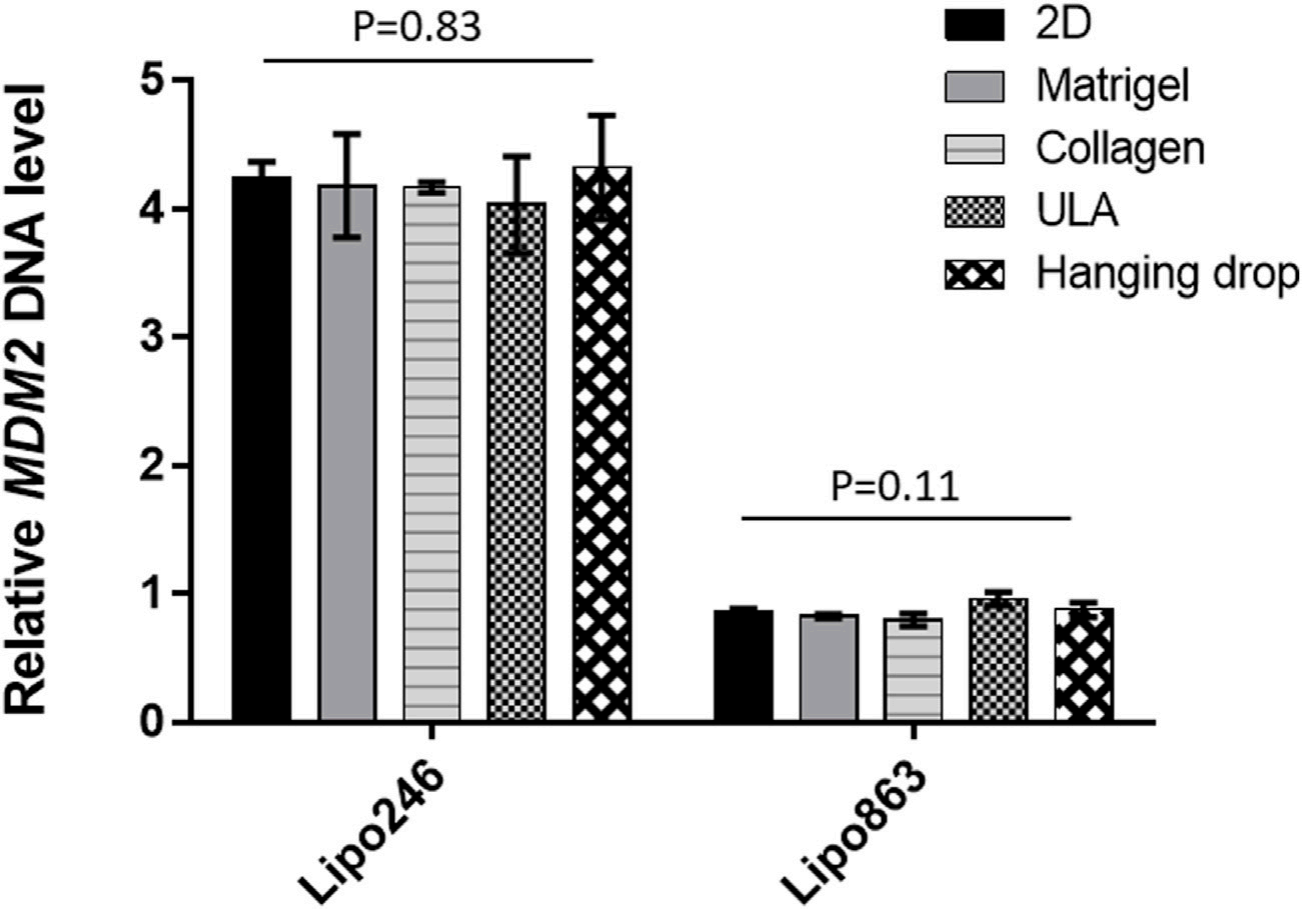
Expression level of *MDM2* DNA determined by qPCR. For both Lipo246 and 863 2D and 3D models, *MDM2* DNA expression levels were determined (2D/Matrigel/Collagen/ULA/Hanging drop) by qPCR analysis. *MDM2* DNA levels were normalized to *GAPDH*. One-way ANOVA was performed for statistical analysis.

The relative *MDM2* DNA level was also assessed by qPCR within 3D models established with all the mentioned techniques. Results were normalized to *GAPDH*. Lipo863 cells showed significantly lower *MDM2* DNA levels than Lipo246 (*p*-value <0.001), which was consistent with our previous results (Bill et al., 2016) and the characteristics of these cell lines (Figure 6). Moreover, we did not observe any significant difference in *MDM2* DNA expression within the same cell line by comparing different 3D models (2D vs. Matrigel vs. Collagen vs. ULA vs. Hanging drop) for both cell lines, *p* = 0.83 in Lipo246 and *p* = 0.11 in Lipo863). We concluded that the relative *MDM2* DNA level of each DDLPS cell line is maintained even after being cultivated in 3D.

### 3.5 Both Lipo246 and 863-collagen 3D models were sensitive to SAR405838 treatment

**FIGURE 7:**
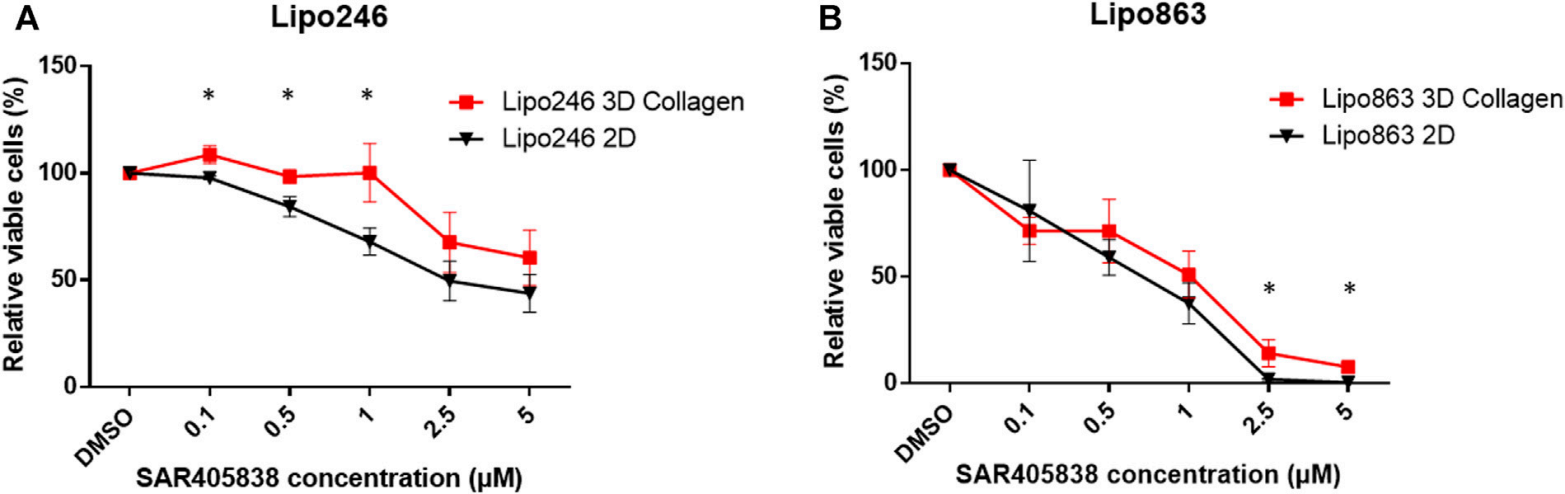
Drug response -Cell Viability Assay- Dose-response curves of Lipo246. **(A)** and Lipo863 **(B)** cultured in 2D or 3D collagen and treated with different concentrations of SAR40538 for 72 h. The relative viable cell percentage of each group was normalized to the control group (DMSO) and ranged between 150% and 0%. Three independent experiments were performed for this assay. The bars are indicating mean ± SD. Student’s *t*-tests was performed for statistical analysis. *p < 0.05.

**FIGURE 8:**
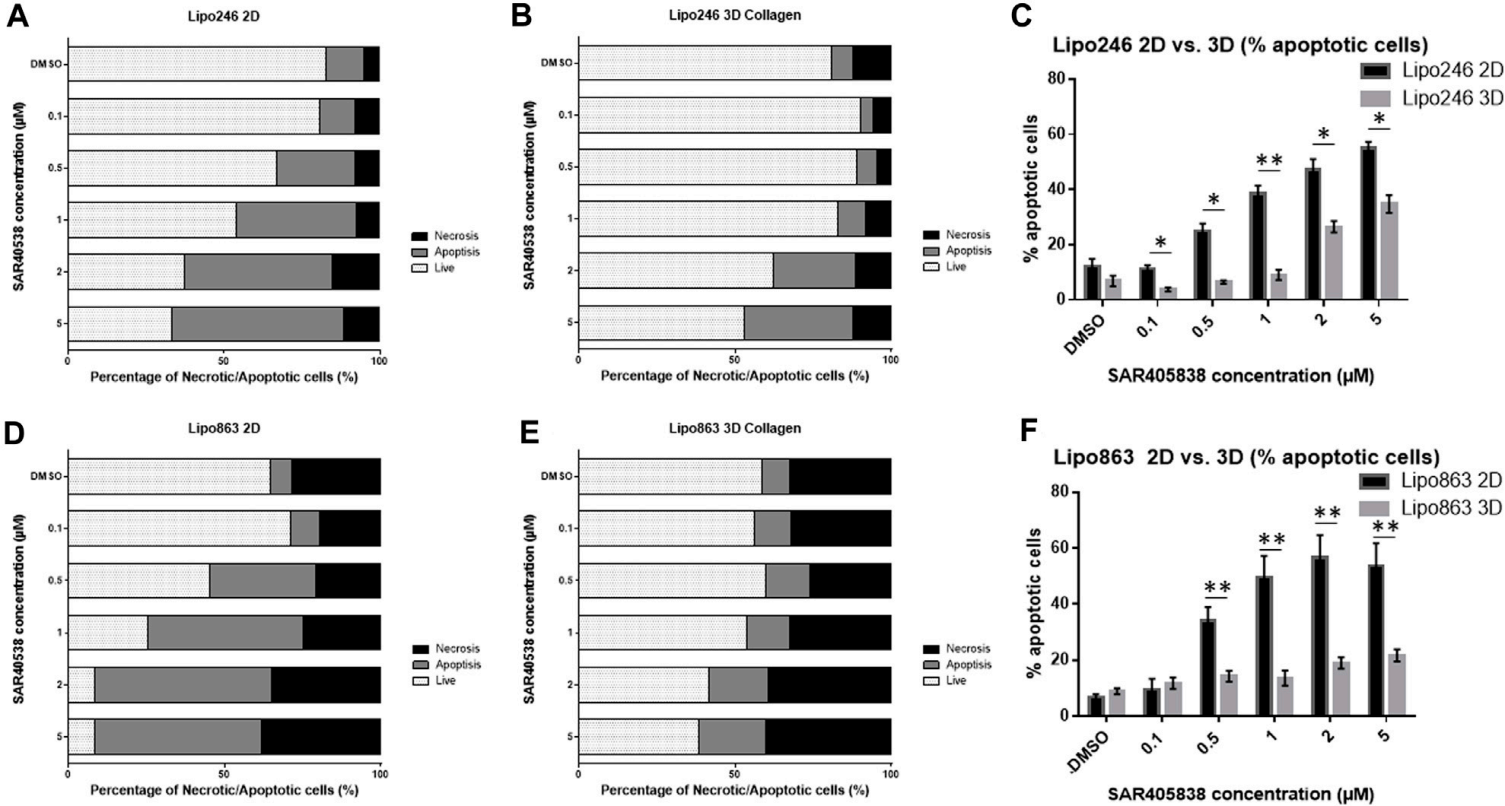
Drug response -Annexin V/PI assay-Graph bars showing the percentage of living cells and cells undergoing necrosis/apoptosis in Lipo246 2D. **(A)**, Lipo246 3D collagen **(B)**, Lipo863 2D **(D)** and Lipo863 3D collagen **(E)**, respectively. Apoptotic cell percentages were compared between 2D and 3D conditions of Lipo246 **(C)** and Lipo863 **(F)**. Three independent experiments were performed for this assay. For statistical analysis, Student’s *t*-test was performed. *p < 0.05, **<0.005.

2D and 3D models (Collagen ECM method) of both Lipo246 and Lipo863 cell lines were treated with DMSO (control) or increasing doses of MDM2 inhibitor SAR405838 (0.1, 0.5, 1, 2, 5 µM) and cell viability was assessed after 72 h. The efficacy of increasing doses of MDM2 inhibitor SAR405838 on our liposarcoma cell lines was already reported (Bill et al., 2016; Tahara et al., 2023), the aim of this assessment was to investigate how our Collagen 3D models were affected by the drug treatment. As shown in Figure 7; Supplementary Figures S4, S5, cells grown in the conventional 2D methods showed sensitivity to the drug, cell viability rate normalized to control (DMSO group) resulted in a dose-dependent decrease ranging from 100% to 33.9% (Lipo246) and from 102.8% to 0.32% (Lipo863). In contrast, even though cells cultured in collagen were also affected by the drug treatment, they showed higher stability than cells grown in conventional culture, especially when they were exposed to higher dose of SAR405838 (>0.5 µM). The range of cell viability rates normalized to control (DMSO group) of 3D models through the assay was from 113.1% to 16.0% (Lipo246) and from 100% to 5.6% (Lipo863), and significant differences between 2D and 3D were seen in 0.1 µM, 0.5 µM and 1 µM groups in Lipo246 and 2.5 µM and 5 µM in Lipo863 when 2D models were compared to paired 3D models (all *p* < 0.05). We also applied the same experimental model to an Annexin V/PI assay to verify whether cells were committed to programmed cell death under the treatment with the same drug. As shown in Figure 8; Supplementary Figures 6, 7, SAR treatment promoted cell death via apoptosis in 2D cell culture models, and the proportion of apoptotic cells dramatically increased in a dose-dependent manner, ranging from 7.7% to 58.9% (Lipo246) and from 6.1% to 65.9% (Lipo863). On the other hand, in the 3D cell culture model the increase of dose-dependent apoptosis was less explicit compared to 2D, ranging from 2.5% to 41% in Lipo246% and 7.2%–25.9% in Lipo863. Comparison between 2D and 3D cells regarding the percentage of apoptotic cells revealed that both Lipo246 and 863 treated with a drug concentration range of 0.1–5 µM showed significant differences, with 2D cultivated groups displaying a significantly higher apoptotic cell population than the corresponding 3D Collagen models (Figures 8C, F).

## 4 Discussion

In this study, we established 3D models from DDLPS patient-derived cell lines using four different methods: Matrigel^®^ ECM and Collagen ECM as scaffold-based methods, ULA plate and Hanging drop as scaffold-free methods. We summarized the advantages and disadvantages of each method in Table 2. Briefly, scaffold-based methods enabled us to investigate cell-ECM interactions by providing a very reliable *in vivo*-mimicking microenvironment, however protocols tended to be more elaborated and expensive. Scaffold-free protocols, on the other hand, were simple and more straightforward with lower costs, but did not allow a proper assessment of cell-ECM interactions, which constitutes a limitation for rigorous investigation of tumor biology.

**TABLE 2:** Advantages and Disadvantages of four different 3D cell culture methods.

		Advantage		Disadvantage	
Matrigel ECM	Scaffold-based	Cell-ECM interaction assessment	Composed of various elements mimicking native ECM	Elaborated protocol	Batch-to-batch variation
		*In vivo*-like microenviroment		Higher cost compared to scaffold-free techniques	Too many undetermined components
Collagen ECM		Long-term culture possibility	Well-determined single components	Additional step is required to isolate cells	Additional step is required to neutralize collagen
		Co-culture possibility	Variation possibility depending on tumor	Low throughput	Optimization is required to mimic native ECM
ULA plate	Scaffold-free	Easy to follow protocol	Uniformed spheroids	No cell-ECM interactions	Higher cost compared to Hanging drop
			One spheroid per well	Obtained results might not be easily related to *in-vivo* conditions	
			Cell growth, treatment and assay in the same well	Unsuitable for long-term culture	
Hanging drop		Gravity-based spheroid	Cost-effective		Transfer step is required for further assessments
			Can be performed with any plate, not required a specific plate or gel		Difficulty for media change

More than 100 types of matrices and scaffolds of both organic and inorganic nature are currently available as substitutes for the ECM (Ravi et al., 2015) for 3D model studies. The ECM provides support to cells, and the mechanical properties of the ECM are determined by the protein content, specifically collagen, laminin, and elastin (McKee et al., 2019). Collagen is a single-component medium having a porous surface with wide gaps that enable other cells to migrate through it (Anguiano et al., 2020). In contrast, Matrigel^®^ is a natural substance that is a reconstituted basement membrane derived from extracts of Engelbreth-Holm-Swarm mouse tumors, consisting primarily of collagen type IV, entactin, perlecan (heparan sulfate proteoglycan), laminin, and growth factors (Hill and Sarkar, 2017).

Several studies have reported that cells showed different morphology, gene expression, and invasiveness when cultivated in collagen compared to Matrigel (Carey et al., 2017; Ruud et al., 2020). Carey et al. (2017) cultured MCF-10A epithelial cells in collagen and Matrigel ECM. They found that morphological differences of proliferating cells and multicellular clusters of cells or organoids were matrix-dependent (Carey et al., 2017). Cells cultivated in pure Matrigel formed organoids with acinar structure, while with more collagen and less Matrigel content in ECM, organoids tended to show increasing invasiveness, losing their rounded morphology and becoming stellate and protrusive. Moreover, E-cadherin, N-cadherin, vimentin, fibronectin and snail expression were quantified using real-time RT-PCR comparing Collagen-rich vs. Matrigel-rich ECM models. In cells cultivated with collagen-enriched ECM, E-cadherin was downregulated more than cells in Matrigel-rich ECM, while other markers were upregulated.

Interestingly, in our study, different morphology and protein expression levels were observed by comparing Matrigel^®^ and collagen models, consistent with results reported on epithelial cell studies. One of our cell lines, Lipo863, naturally aggregated and formed spheroids when cultivated in Matrigel^®^. However, the same result was not achieved when the same cells were cultivated in collagen as cells lost their spherical structures. We hypothesized that matrix rigidity could be one of the factors affecting constructs’ morphological structures. Using normal murine mammary gland epithelial cells (NMuMG) and Madin–Darby canine kidney epithelial cells (MDCK), Leight et al. (2012) reported that cells cultivated on the most rigid collagen gels formed cuboidal shapes and sheeted on the surface identical to cells on tissue culture plastic. In contrast, cells on compliant gels showed more round shape and formed spherical clusters (Leight et al., 2012). Shintani et al. (2008) reported that cells plated on collagen I showed several mesenchymal marker upregulations, including N-cadherin, vimentin, smooth muscle actin, and fibronectin, while E-cadherin was downregulated, concluding that collagen type I was capable of inducing an epithelial-mesenchymal transition (EMT)-like response in lung adenocarcinoma cell line A549 (Shintani et al., 2008). Most of research evaluating the effects of different scaffolds in 3D cultures was conducted using epithelial cells, while few achievements have been accomplished using mesenchymal cells, including liposarcoma, and many questions are still unanswered. For example, we need to understand the scaffold components, not only the main ingredients such as Matrigel or collagen, but also the concentration of specific components as well as the rigidity of gels, as they might lead to different consequences, hence affecting our results. Therefore, a better understanding of scaffold characteristics is needed for an ideal 3D cell culture design to better enable further investigations, such as whole genome sequencing, protein sequencing and pathway analysis should be able to reveal those differences.

The higher drug resistance of 3D models compared to 2D models has been reported in both scaffold-free and scaffold-based techniques. Lv et al. (2016) reported that 3D glioma culture constructs embedded in collagen showed higher resistance to temozolomide, lomustine, and cisplatin compared to 2D culture constructs. Imamura et al. (2015) studied drug resistance using breast cancer 2D model and 3D spheroids generated by scaffold-free method and reported that dense/bigger spheroids tended to show higher resistance to paclitaxel and doxorubicin treatment, whereas small/looser spheroids were as sensitive to the drugs as 2D samples. Breslin and O’Driscoll concluded that the biological information represented by 3D and 2D cell cultures is substantially different, i.e., 3D cell cultures tend to demonstrate an increase in resistance to anti-cancer drugs compared to 2D cultures, and this may be facilitated by altered receptor proteins, drug transporters and metabolizing enzyme activity (Breslin and O’Driscoll, 2016). Numerous anticancer drugs were eliminated during clinical therapeutic development, indicating that a 2D-culture-based screening platform may overreact for anticancer drug responses and therefore unable to precisely mimic tumor conditions *in vivo*. Drug resistance comparison between 2D and 3D models derived from liposarcoma cell lines are not yet effectively addressed so far, and further investigation is required for a better understanding leading to discover more effective therapies.

Recent reports have shown promising results for the application of 3D cell culture in collagen. Forsythe et al. (2022) successfully cultivated several sarcoma cells derived from patients using collagen as one of the ECM, generating organoids that were subsequently applied for the assessment of personalized treatments. In another study, Lin utilized collagen as an ECM component to culture spheroids using murine primary pancreatic cancer cell lines and successfully conducted an invasion assay (Lin et al., 2020). These findings highlight the potential of collagen as a scaffold for 3D cell culture and its potential application in various research areas, including cancer research. One of our future key goals is advancing this methodology by integrating it with a microfluidic device, which would allow us to explore the interaction between cells and the TME in a more comprehensive manner (Akbari et al., 2018). Previous research has shown that microfluidic devices can better preserve extracellular vesicle (EV) cargo during the isolation of particles (Perut et al., 2019; Casadei et al., 2021), providing us with a unique opportunity to gain insights into the mechanisms underlying DDLPS recurrence events. Previously, we have observed high levels of *MDM2* DNA in EVs derived from DDLPS patient serum and cell lines, which was transferable to preadipocytes at the microenvironment level (Casadei et al., 2019; Casadei and Pollock, 2020). Building on this observation, we hypothesize that the DDLPS microenvironment plays a crucial role in DDLPS recurrence. Specifically, we propose that *MDM2* DNA-rich EVs are transferred from DDLPS to adjacent or remote non-tumor tissue and that these EVs trigger tumor progression. We believe that utilizing a microfluidic device in conjunction with 3D collagen models can allow us to elucidate the mechanical role of EVs more effectively as one of the components in the tumor microenvironment of DDLPS. This approach represents a significant step forward in enabling a deeper understanding of DDLPS biology and the development of effective therapies.

## Data Availability

The raw data supporting the conclusion of this article will be made available by the authors, without undue reservation.
