# Supplementary figures and images for: Comparison of three-dimensional cell culture techniques of dedifferentiated liposarcoma and their integration with future research
Source: Front Cell Dev Biol. 2024 Mar 4;12:1362696. doi: 10.3389/fcell.2024.1362696 (PMC10945377; doi:10.3389/fcell.2024.1362696)

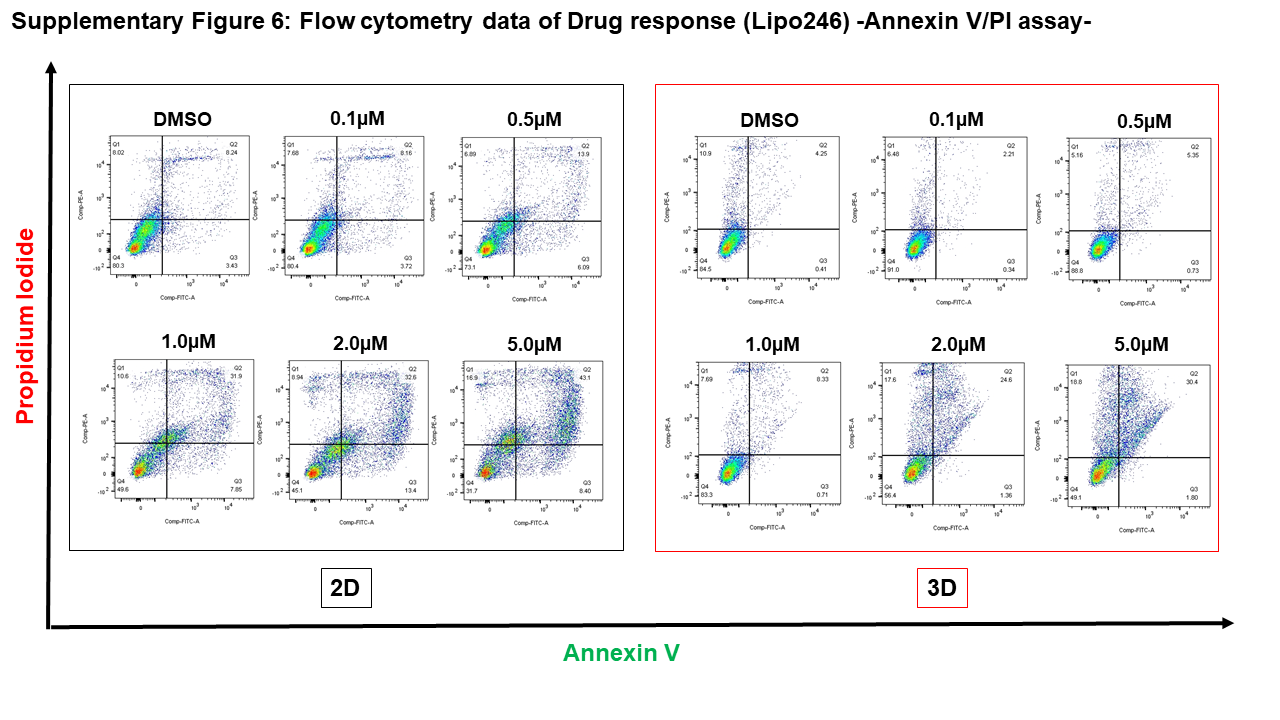

Supplement: Supplementary file 1 [file Image6.TIF]

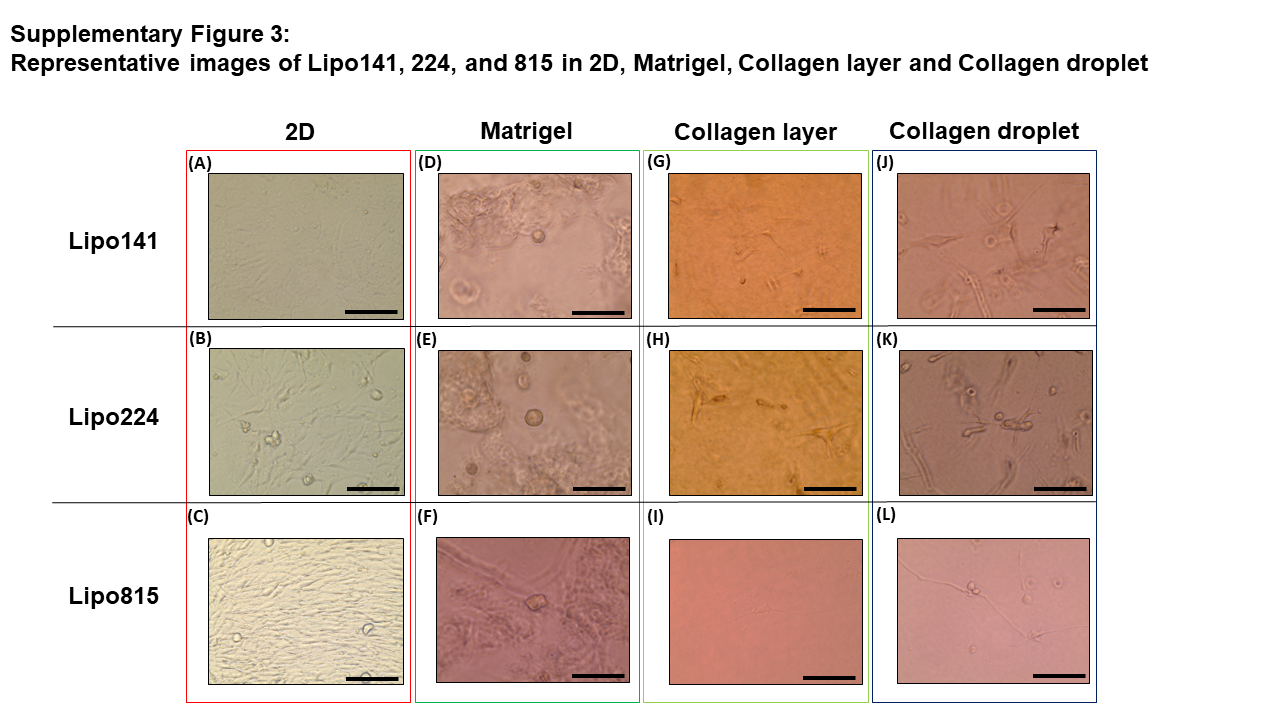

Supplement: Supplementary file 2 [file Image3.TIF]

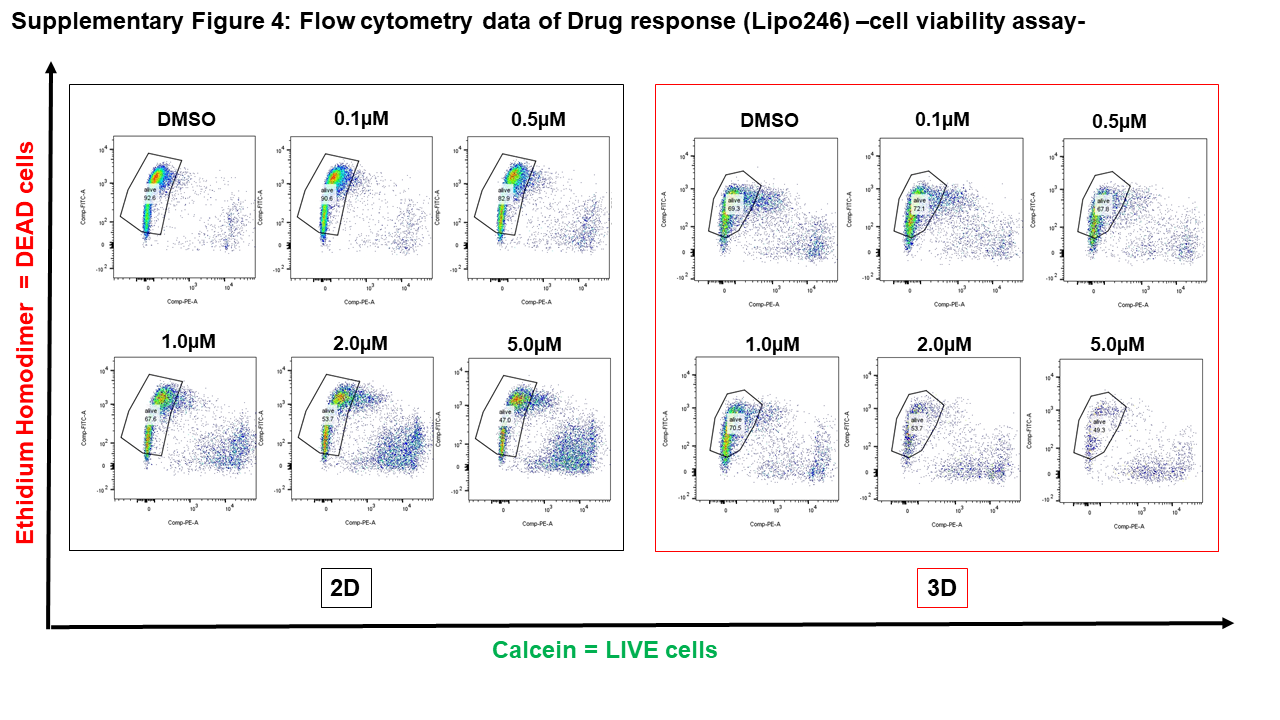

Supplement: Supplementary file 3 [file Image4.TIF]

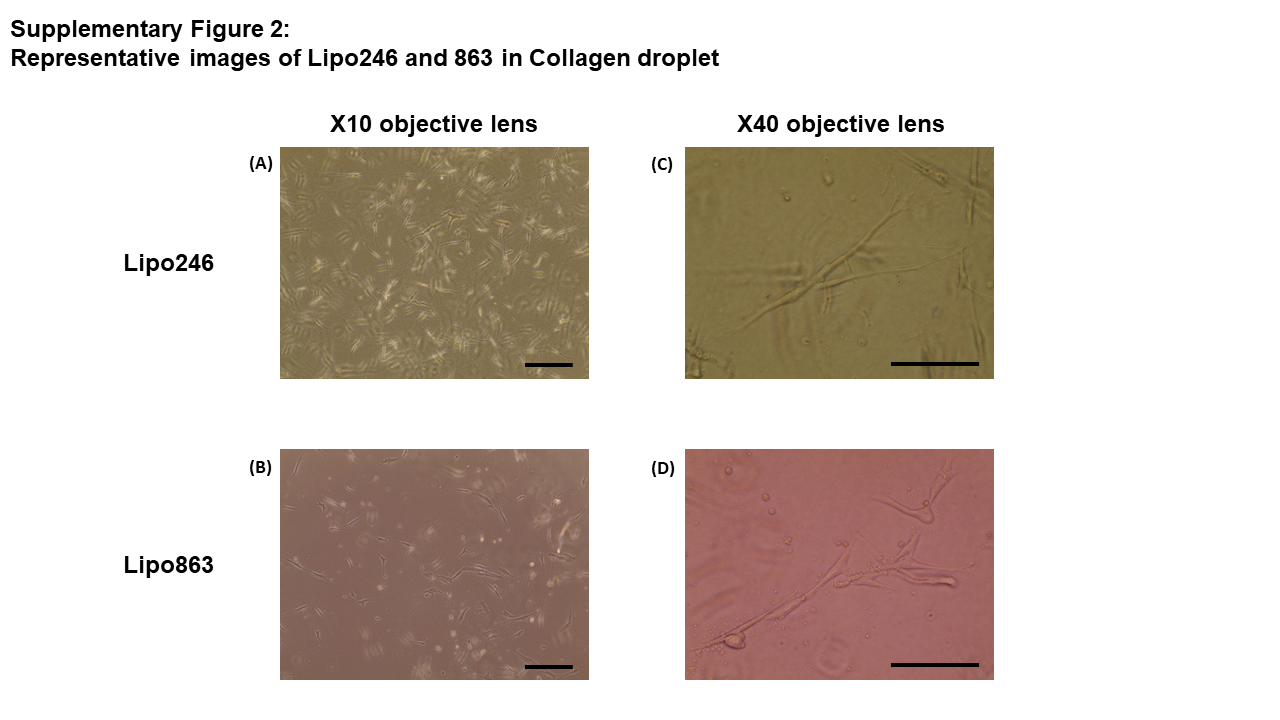

Supplement: Supplementary file 4 [file Image2.TIF]

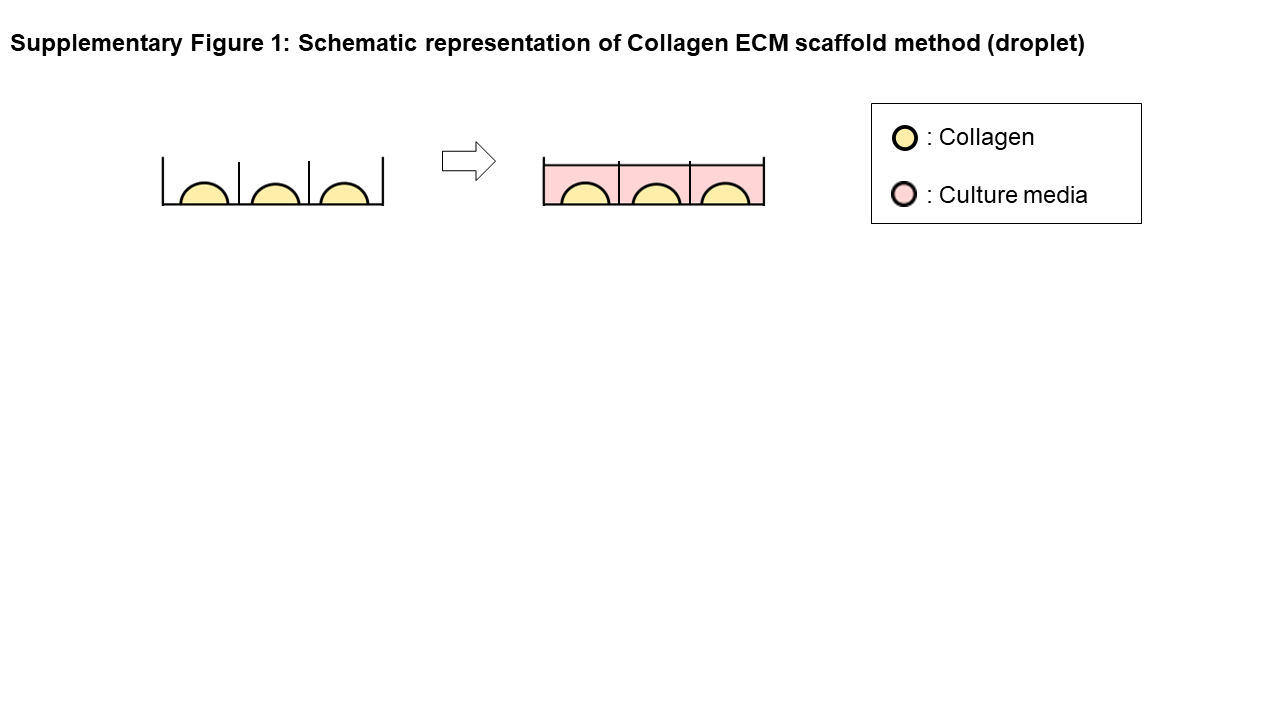

Supplement: Supplementary file 5 [file Image1.TIF]

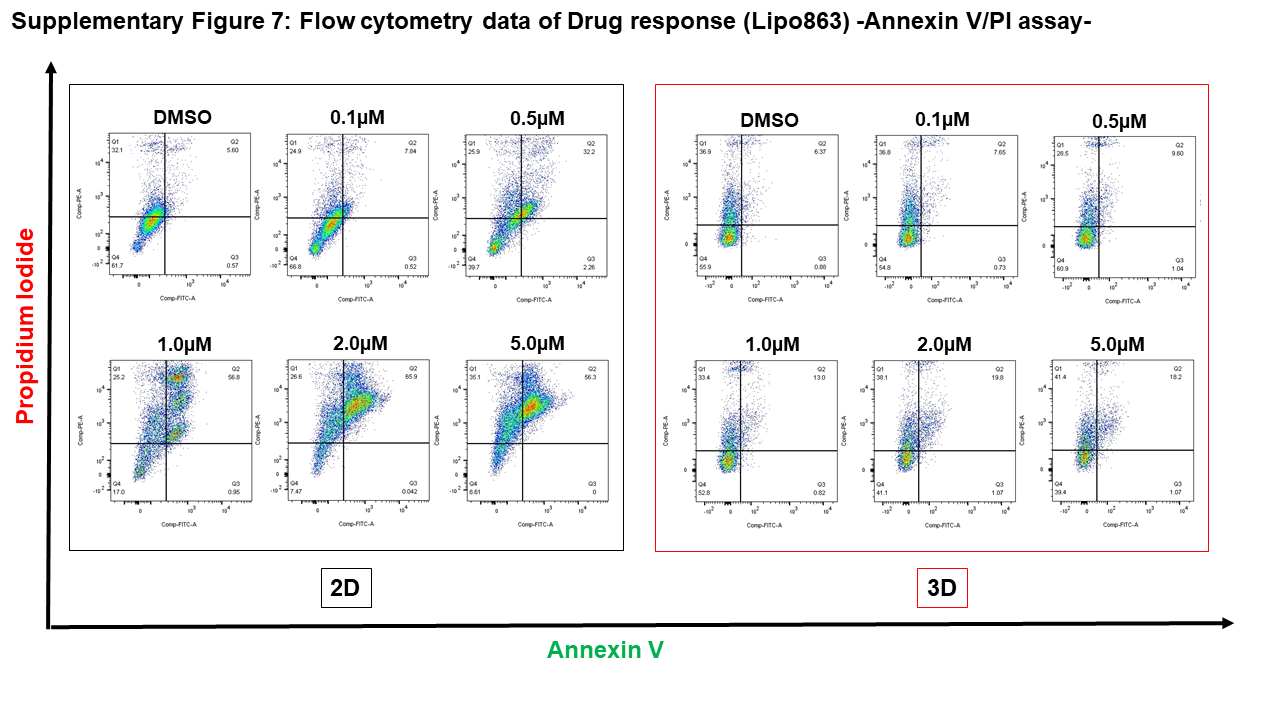

Supplement: Supplementary file 6 [file Image7.TIF]

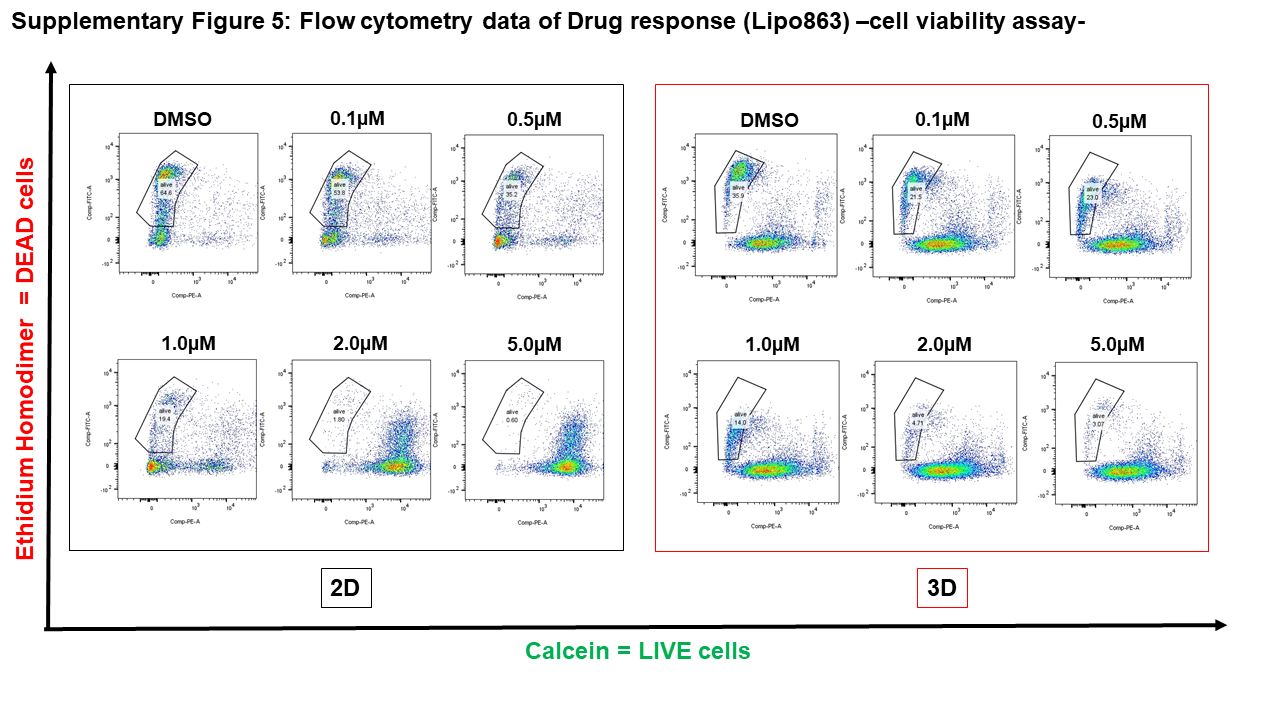

Supplement: Supplementary file 8 [file Image5.TIF]
