# Supplementary material for: Comparison of three-dimensional cell culture techniques of dedifferentiated liposarcoma and their integration with future research
Source: Front Cell Dev Biol. 2024 Mar 4;12:1362696. doi: 10.3389/fcell.2024.1362696 (PMC10945377; doi:10.3389/fcell.2024.1362696)
Supplement: Supplementary file 7 [file DataSheet1.pdf]

<Figure legend for Supplementary figure>

Supplementary Figure 1: Schematic representation of Collagen ECM scaffold method (droplet)
Seed 50µl/well of the mix of single-cell suspension and collagen solution into a 12-well plate and incubate at 37°C for 30 minutes to solidify the mixture -> add 500µl of culture media.

Supplementary Figure 2: Representative images of Lipo246 and 863 in Collagen droplet
Low magnification (X10 objective lens) and high magnification (X40 objective lens) images. Neither Lipo246 nor 863 formed spheroids, showing spindle shaped single cell distribution, partially bridging to each other. Scale bar is indicating 250 µm in (A, B), and 125 µm in (C, D).

Supplementary Figure 3: Representative images of Lipo141, 224, and 815 in 2D, Matrigel, Collagen layer and Collagen droplet
(A-C) 2D cell culture, (D-F) Matrigel® ECM scaffold method 3D cell culture, (G-I) Collagen ECM scaffold method (layer) 3D cell culture, (J-L) Collagen ECM scaffold method (droplet) 3D cell culture, respectively. All images were taken with 40x objectives, scale bar is indicating 125µm.

Supplementary figure 4: Flow cytometry data of Drug response (Lipo246) –cell viability assay-
Cell viability of Lipo246 was analyzed by flow cytometry using Invitrogen™ LIVE/DEAD™ Viability/Cytotoxicity Kit for mammalian cells. 2D and 3D collagen samples were treated with DMSO or increasing doses of MDM2 inhibitor SAR405838 (0.1, 0.5, 1, 2, 5 µM) for 72 hours.

Supplementary figure 5: Flow cytometry data of Drug response (Lipo863) –cell viability assay-
Cell viability of Lipo863 was analyzed by flow cytometry using Invitrogen™ LIVE/DEAD™ Viability/Cytotoxicity Kit for mammalian cells. 2D and 3D collagen samples were treated with DMSO or increasing doses of MDM2 inhibitor SAR405838 (0.1, 0.5, 1, 2, 5 µM) for 72 hours.

Supplementary figure 6: Flow cytometry data of Drug response (Lipo246) –Annexin V/PI assay-
Cell apoptosis of Lipo246 was analyzed by flow cytometry using TACS® Annexin V-FITC Apoptosis Detection kit. 2D and 3D collagen samples were treated with DMSO or increasing doses of MDM2 inhibitor SAR405838 (0.1, 0.5, 1, 2, 5 µM) for 72 hours.

Supplementary figure 7: Flow cytometry data of Drug response (Lipo863) –Annexin V/PI assay-

Cell apoptosis of Lipo863 was analyzed by flow cytometry using TACS® Annexin V-FITC Apoptosis Detection kit. 2D and 3D collagen samples were treated with DMSO or increasing doses of MDM2 inhibitor SAR405838 (0.1, 0.5, 1, 2, 5 μM) for 72 hours.
